# Effects of sacubitril/valsartan on both metabolic parameters and insulin resistance in prediabetic non-obese patients with heart failure and reduced ejection fraction

**DOI:** 10.3389/fendo.2022.940654

**Published:** 2022-08-10

**Authors:** Cosima Cloro, Isabella Zaffina, Luca Sacchetta, Federico Arturi, Cristina Clausi, Stefania Lucà, Maria Chiara Pelle, Federica Giofrè, Giuseppe Armentaro, Valentina Forte, Francesco Mario De Rosa, Angela Sciacqua, Franco Arturi

**Affiliations:** ^1^ Unit of Cardiology, Annunziata Hospital, Cosenza, Italy; ^2^ Unit of Internal Medicine, Department of Medical and Surgical Sciences, University of Magna Graecia, Catanzaro, Italy; ^3^ Department of Clinical and Experimental Medicine, University of Pisa, Pisa, Italy; ^4^ Unit of Cardiology, University of Padova, Padova, Italy; ^5^ Unit of Endocrinology, University of Padova, Padova, Italy; ^6^ Geriatric Unit, Department of Medical and Surgical Sciences, University of Magna Graecia, Catanzaro, Italy; ^7^ Research Center for the Prevention and Treatment of Metabolic Diseases (CR METDIS), University of Magna Graecia, Catanzaro, Italy

**Keywords:** prediabetes, type 2 diabetes, cardiovascular disease, insulin, beta cell, HFrEF, sacubitril/valsartan, insulin resistance

## Abstract

**Background:**

The effects of sacubitril/valsartan (sac/val) on metabolic parameters and insulin resistance (IR) in non-obese/prediabetic patients have not been previously described.

**Aim:**

To evaluate the effects of sac/val on glycemic and metabolic parameters, Homeostatic Model Assessment of IR (HOMA-IR), and echocardiographic parameters in prediabetic patients with heart failure with reduced ejection fraction (HFrEF).

**Methods:**

Fifty-nine patients with HFrEF (EF < 35%) but without obesity and/or type 2 diabetes mellitus have been enrolled. All the patients at baseline and week 24 underwent complete anthropometrical evaluation and were subjected to an echocardiogram test. IR has been assessed by HOMA-IR.

**Results:**

After 24-week of treatment with sac/val, a significant reduction in fasting plasma glucose (109 ± 9 *vs* 103 ± 8 mg/dl, p < 0.0001), fasting plasma insulin (16 ± 4 *vs* 10 ± 4 UI/L), and hemoglobin A1c (HbA1c) value (6% ± 0.5% *vs* 5.3% ± 0.3%, p < 0.0001) was observed. Similarly, we observed a significant improvement in IR (HOMA-IR, 4.4 ± 0.9 *vs* 2.5 ± 0.6, p < 0.0001). The echocardiogram evaluation showed a significant reduction of the left ventricular end-diastolic volume (168 ± 24 *vs* 158 ± 22 ml, p < 0.05), a significant reduction of the left ventricular end-systolic volume (111 ± 26 *vs* 98 ± 22 ml, p < 0.005), and a significant reduction of E/e′ ratio. Sac/val use was also associated with an average 5.1% increase in ejection fraction.

**Conclusions:**

Our data seem to indicate that sal/val enhances metabolic control and improves insulin resistance also in prediabetic non-obese patients with HFrEF.

## Introduction

Heart failure (HF) affects at least 26 million people worldwide, and its prevalence is steadily increasing (1). Despite important achievements in pharmacological treatment, HF is still characterized by high morbidity and mortality. Even if beta-blockers (BBs) and renin–angiotensin–aldosterone system (RAAS) inhibitors represent the cornerstone of pharmacological therapy for HF with reduced ejection fraction (HFrEF), new therapeutic targets have been identified to improve clinical outcomes. Sacubitril/valsartan (sac/val) is an angiotensin receptor-neprilysin inhibitor (ARNI) approved in Italy in March 2017, which combines selective AT1-receptor blockade with reduced degradation of natriuretic peptides (NPs) exerting positive effects on the cardiovascular (CV) system (2). In the PARADIGM-HF clinical trial, it has been demonstrated that sac/val was able to reduce the composite endpoint of CV death or first hospitalization for HF by 20% and the relative risk of all-cause mortality by 16% in comparison with enalapril, after a median follow-up of 27 months, in HFrEF outpatients (3).

HF often occurs simultaneously with type 2 diabetes mellitus (T2DM), and each disease independently increases the risk for the other. Several studies demonstrated that individuals with T2DM have a greater risk for HF than those without the disease (4), and diabetes is considered to be an independent risk factor for the progression of HF with preserved or reduced ejection fraction and major comorbidity (5). Insulin resistance (IR) and metabolic alterations are present in patients with HF (6). T2DM can lead to the development of HF *via* systemic, myocardial, and cellular pathways (4). Hyperglycemia and hyperinsulinemia facilitate the process of atherosclerosis by promoting smooth vascular muscle cell proliferation and inducing inflammation (4). Patients with T2DM often develop diastolic or systolic dysfunction without other obvious causes of cardiomyopathy, a condition defined as diabetic cardiomyopathy, and IR directly promotes myocardial hypertrophy (4, 7). Furthermore, the prediabetic condition seems to be associated with a substantially increased risk of adverse outcomes in patients with HFrEF.

IR is also common in non-diabetic patients with HF and has been associated with adverse prognosis (8); however, the pathogenetic mechanisms that link IR to unfavorable clinical outcomes in non-diabetic HF patients are not completely understood. Nevertheless, several evidence suggests that in patients with HF, impaired insulin sensitivity is related to higher mortality, independently of body composition and other well-established risk factors, and might have implications for the pathophysiology of HF disease progression (8). Thus, therapeutically targeting impaired insulin sensitivity could potentially be beneficial in patients with HF. In the *post-hoc* analysis of the PARADIGM-HF clinical study, in patients with T2DM, a greater reduction of hemoglobin A1c (HbA1c) concentrations in patients treated with sac/val than in those taking enalapril has been demonstrated (9).

On the contrary, at present, there are no data on the effects of sac/val on IR, fasting plasma glucose, fasting plasma insulin, or other metabolic parameters in prediabetic non-obese patients with HF and reduced ejection fraction (EF < 35%).

In this study, we evaluated the effects of sac/val on IR, metabolic, and echocardiographic parameters in prediabetic non-obese patients with HF and reduced ejection fraction (EF < 35%).

## Materials and methods

The study population consisted of consecutive outpatients referring to the Cardiology Unit, of the ‘Annunziata’ Hospital, Cosenza, Italy, and to the Internal Medicine Unit, of the University Hospital of Catanzaro, Italy. A total of 59 patients have been enrolled (38 men and 21 women, average age 64.5 ± 6.5 years). Eligibility criteria for the study were a diagnosis of HFrEF (EF < 35%), New York Heart Association (NYHA) II–III, persistence of symptoms despite an optimized treatment with stable doses of angiotensin-converting enzyme inhibitors (ACE-Is) or angiotensin receptor blockers (ARBs) for at least 4 weeks, absence of both, obesity (body mass index (BMI) ≥30 kg/m^2^), T2DM [fasting plasma glucose (FPG) ≥126 mg/dl (7.0 mmol/L), hemoglobin A1c (HbA1C) ≥6.5% (48.1 mmol/mol), or 2-h plasma glucose (2-h PG) value ≥200 mg/dl (11.1 mmol/L) during oral glucose tolerance test (OGTT), and current treatment with anti-diabetic drugs or self-reported history of a previous diagnosis) (10)], and age >18 years. The main exclusion criteria included any prior echocardiographic measurement of left ventricular ejection fraction (LVEF) >35%, recent acute coronary syndrome, acute decompensated HF at the time of screening, cardiac surgery or percutaneous coronary intervention, intolerance to either study drug (or similar classes) or a history of angioedema, systolic blood pressure (BP) >180 or <110 mmHg, estimated glomerular filtration rate (eGFR) <30 ml/min/1.73 m^2^, and serum potassium >5.2 mmol/L.

At baseline and then after 24 weeks of treatment, all the participants underwent a complete anthropometrical evaluation, including BMI, waist circumference, heart rate assessment, and three consecutive measurements of clinic blood pressure obtained in the sitting position, after 5 min of quiet rest. All subjects underwent laboratory determinations, including glucose-related parameters [HbA1c, FPG, and fasting plasma insulin], cardiovascular biomarkers (N-terminal pro-brain natriuretic peptide (NT-proBNP levels)), lipids [total cholesterol, low-density lipoprotein cholesterol (LDL)-cholesterol, high-density lipoprotein cholesterol (HDL)-cholesterol, hematology, and biochemistry including amylase, lipase, and creatinine]. The IR has been assessed by Homeostatic Model Assessment (HOMA)-IR.

A 75-g OGTT was performed as previously described (10) for patients with FPG ≥ 100 but <126 mg/dl [impaired fasting glucose (IFG)] and HbA1c value >6% but <6.5%, after a 12-h overnight fast.

At baseline and then at 24 weeks of treatment, all participants underwent an electrocardiogram (ECG) and echocardiogram tests. The Minnesota Living with HF questionnaire was used to evaluate the quality of life (11), and the determination of NYHA functional class was performed as suggested by the European Society of Cardiology (ESC) guidelines for the diagnosis and treatment of acute and chronic HF (12).

For eligible patients with sac/val therapy discontinued, a 75-g OGTT was performed as previously described (10), including ACE-I (at least 36 h before) or ARB, and received initial dosages of 24/26 or 49/51 mg b.i.d. according to their clinical conditions. Moreover, sac/val dosage was increased every 2–4 weeks up to the maximum tolerated dose, as recommended.

The patients enrolled showed persistence of symptoms despite an optimized treatment with beta-blockers, mineral receptor antagonists, loop diuretic, and stable doses of ACE-Is or ARBs for at least 4 weeks.

In our study, 43 patients (73%) were previously in treatment with ACE inhibitors, 16 (27%) were treated with ARB, and only 7 (12%) were treated with valsartan.

The study was approved by the local ethics committees, and informed written consent was obtained from all the participants before the initiation of the study. The study was conducted in accordance with the ethical rules of the Declaration of Helsinki.

### Biochemical assays

Plasma glucose, total and HDL cholesterol, triglycerides, and uric acid concentrations were measured by enzymatic methods (Roche Diagnostics, Mannheim, Germany). Plasma insulin concentration was determined by a chemiluminescence-based assay (Roche Diagnostics, Mannheim, Germany). HbA1c was measured with high-performance liquid chromatography using a National Glycohemoglobin Standardization Program (NGSP)-certified automated analyzer and IFCC (Adams HA-8160 HbA1C analyzer, Menarini, Italy; normal reference range, 4.3%–5.9%). Serum creatinine was measured in the routine laboratory by an automated technique based on a creatinine Jaffè compensated method for serum and plasma (Roche, Basel, Switzerland) method implemented in an auto-analyzer. All other metabolites were measured by standard methods.

Moreover, HOMA-IR was determined using the following simplified equation:


HOMA−IR=(Fasting Plasma Insulin×Fasting Plasma Glucose)/22.5.

### Echocardiographic parameters

Tracings were taken with the patient in partial left decubitus position, using a VIVID 7 Pro ultrasound machine (GE Technologies, Milwaukee, WI, USA) with an annular phased array 2.5-MHz transducer. Echocardiographic readings were made in random order by the investigator who had no knowledge of patients’ BP and other clinical data. Only frames with optimal visualization of cardiac structures were considered for reading. The mean values from at least four measurements of each parameter for each patient were computed. As previously reported, subjects having a left ventricular ejection fraction >35% were excluded from this study. The measurements were obtained according to the international guidelines (13). Left ventricular end-diastolic volume (LVEDV) and left ventricular end-systolic volume (LVESV) were calculated according to the biplane Simpson’s method, and the LVEF was calculated according to the following formula:


LVEF=LVEDV−LVESV/LVEDV∗100

as mean of two measures in four and two apical chambers.

Measurements of interventricular septum (IVS) thickness, posterior wall (PW) thickness, and left ventricular internal diameter (LVID) were made at end-diastole and end-systole. Left ventricular diastolic function was evaluated according to diagnostic criteria proposed by the American Society of Echocardiography (14). Evaluation of left atrial volume (LAV) was obtained using the apical four-chamber and two-chamber views. A pulsed Doppler transmitral flow velocity profile was obtained from the apical four-chamber view, and the sample volume was positioned at the tip of the mitral valve leaflets. The following parameters were evaluated for diastolic function: E wave (peak transvalvular flow velocity in early diastole), A wave (peak transvalvular flow velocity in late diastole), E/A ratio, and E/A ratio between 1 and 2 was defined as normal.

Pulsed wave tissue Doppler imaging (TDI) was performed at the junction of the septal and lateral mitral annulus. Early diastolic (septal e′ and lateral e′) and late diastolic (septal a′ and lateral a′) velocities were recorded; the ratio of E/e′ (average) was also calculated.

### Statistical analysis

Within each group, a paired Student’s t-test was used to compare means between baseline and 24 weeks. Categorical variables were compared by the χ^2^ test. Data are reported as means ± SD. A p-value <0.05 was considered statistically significant. All analyses were performed using the SPSS software program Version 23.0 for Windows.

## Results

A total of 59 prediabetic patients (38 men and 21 women, average age of 64.5 ± 6.5 years) and non-obese (BMI 26.4 ± 2.6 kg/m^2^) with HFrEF were evaluated. Thirty-seven of the 59 patients (62.7%) were in the NYHA III class, and 22 (37.2%) were in the NYHA II class. Twenty-one patients had normal glucose tolerance (NGT), 29 had IFG, and nine had impaired glucose tolerance (IGT) after OGTT according to the American Diabetes Association (ADA) guidelines (10). Twenty-two of the 59 patients (38%) were prescribed sac/val at a 24/26 mg dosage, 26 of the 59 patients (44%) assumed a dose of 49/51 mg, and 11 (18%) assumed a 97/103 mg dosage. The most frequent comorbidities in the study population were ischemic heart disease (79.6%), atrial fibrillation (32.3%), hypertension (83%), and dyslipidemia (88%) (**Table 1**).

**Table 1:** Baseline characteristics and comorbidities of enrolled patients.

	Baseline
**Variables**	(n = 59)
**Age**, years	64.5 ± 6.5
**Sex**, m/f	38/21
**Ischemic heart disease**, n (%)	47 (79.6)
**Atrial fibrillation**, n (%)	19 (32.2)
**Arterial hypertension**, n (%)	49 (83)
**Dyslipidemia**, n (%)	52 (88)

Data are means ± SD.

Anthropometric, hemodynamic, and biohumoral characteristics at baseline and after 24 weeks of therapy with sac/val are summarized in **Table 2**. No differences in weight, BMI, and waist circumference were observed. The comparison between baseline and 24-week values showed a significant reduction in heart rate (HR) (74.3 ± 2.6 *vs* 70.7 ± 6.5 bpm, p < 0.0001), systolic blood pressure (118.2 ± 7.7 *vs* 112.3 ± 6.7 mmHg, p < 0.0001), and diastolic blood pressure (73.4 ± 7.4 *vs* 68.6 ± 5.7 mmHg, p < 0.0001). Furthermore, there was a significant reduction in serum levels of sodium (140.4 ± 2.6 *vs* 138.6 ± 1.4 mmol/L, p = 0.003) and a significant change in serum levels of potassium (4.5 ± 0.4 *vs* 4.6 ± 0.3 mmol/L, p < 0.0001), while serum levels of creatinine (1.1 ± 0.6 *vs* 1.0 ± 0.7 mg/ml, p = 0.4) were not significantly modified. After 24 weeks of treatment with sac/val, we noticed a significant reduction of NT-proBNP levels (1,732 ± 536 *vs* 883 ± 376 pg/ml, p < 0.0001) and significant improvements in patients’ functional status, as showed by NYHA class changes: class III (from 62.7% to 32.2% of the whole cohort, p < 0.0001) and class II (from 37.2% to 67.7%, p < 0.0001). No differences in serum amylase, serum lipase, serum aspartate aminotransferase (AST), serum alanine aminotransferase (ALT), and high-sensitivity C-reactive protein (hs-CRP) were observed (data not shown).

**Table 2:** Anthropometric, hemodynamic, and biohumoral characteristics at baseline and after 24 weeks of therapy with sac/val.

	Baseline	Follow-up	P Value
Variables	(n = 59)	(n = 59)	
**Weight,** kg	72.6 ± 13	71.8 ± 11	0.7
**BMI**, kg/m^2^	26.4 ± 2.6	26.1 ± 2.2	0.5
**WC**, cm	96 ± 8	95 ± 7	0.5
**SBP**, mmHg	118.2 ± 7.7	112.3 ± 6.7	<0.0001
**DBP**, mmHg	73.4 ± 7.4	68.6 ± 5.7	<0.0001
**HR**, bpm	74.3 ± 2.6	70.7 ± 6.5	<0.0001
**eGFR,** ml/min/1.73 m^2^	67.2 ± 19.2	68.4 ± 22	0.75
**NT-proBNP**, pg/ml	1,732 ± 536	883 ± 376	<0.0001
**MLHFQ**, score	92.5 ± 3.4	86.4 ± 4.5	<0.0001

Data are means ± SD.

BMI, body mass index; WC, waist circumference; SBP, systolic blood pressure; DBP, diastolic blood pressure; HR, heart rate; eGFR, estimated glomerular filtration rate; AST, aspartate aminotransferase; ALT, alanine aminotransferase; hs-CRP, high-sensitivity C-reactive protein; NT-proBNP, N-terminal pro-B-type natriuretic peptide; MLHFQ, Minnesota Living with Heart Failure Questionnaire; sac/val, sacubitril/valsartan.

**Table 3** shows the metabolic characteristics at baseline and after 24 weeks of therapy with sac/val. After 24 weeks of treatment with sac/val, a significant reduction in FPG (109 ± 9 *vs* 103 ± 8 mg/dl, p < 0.0001), fasting plasma insulin (16 ± 4 *vs* 10 ± 4 UI/L), and HbA1c value (6% ± 0.5% *vs* 5.3% ± 0.3%, p < 0.0001) were observed. Similarly, we observed a significant improvement in IR (HOMA-IR, 4.4 ± 0.9 *vs* 2.5 ± 0.6, p < 0.0001). We have also evaluated the metabolic parameters in a group of prediabetic non-obese patients with HFrEF (n = 22) treated with standard therapy (27% in treatment with ARB and 73% in treatment with ACE-i), and no significant differences in metabolic parameters have been found after 24 weeks of treatment (data not shown).

**Table 3:** Metabolic characteristics at baseline and after 24 weeks of therapy with sac/val.

	Baseline	Follow-up	P Value
Variables	(n = 59)	(n = 59)	
**Fasting plasma glucose**, mg/dl	109 ± 9	103 ± 8	<0.0001
**Fasting plasma insulin**, UI/L	16 ± 4	10 ± 4	<0.0001
**HbA1C**, %	6 ± 0.5	5.3 ± 0.3	<0.0001
**HOMA-IR**	4.4 ± 0.9	2.5 ± 0.6	<0.0001
**Total cholesterol,** mg/dl	194 ± 30	161 ± 28	<0.0001
**HDL-cholesterol**, mg/dl	50 ± 12	51 ± 10	0.6
**LDL-cholesterol**, mg/dl	116 ± 32	82 ± 27	<0.0001
**Triglycerides**, mg/dl	137 ± 58	136 ± 46	0.9
**Serum UA**, mg/dl	5.9 ± 0.6	4.8 ± 1.0	<0.0001

Data are means ± SD.

HbA1C, glycated hemoglobin A1C; HOMA-IR, Homeostatic Model Assessment of insulin resistance; HDL-cholesterol, high-density lipoprotein cholesterol; LDL-cholesterol, low-density lipoprotein cholesterol; serum UA, uric acid; sac/val, sacubitril/valsartan.

Furthermore, in patients treated with sac/val, there was a significant change in serum levels of total cholesterol (194 ± 30 *vs* 161 ± 28 mg/dl, p < 0.0001) and LDL-cholesterol (116 ± 32 *vs* 82 ± 27 mg/dl, p < 0.0001) but not in serum levels of HDL-cholesterol and serum levels of triglycerides (**Table 3**). There was also a statistically significant decrease in serum uric acid (5.9 ± 0.6 *vs* 4.8 ± 1.0 mg/dl, p < 0.0001).

The echocardiogram evaluation showed a significant reduction of the LV diastolic volume (LVDV) (168 + 24 *vs* 158 + 22 ml, p < 0.05), a significant reduction of the LV systolic volume (LVSV) (111 + 26 *vs* 98 + 22 ml, p < 0.005), and a significant reduction of E/e′ ratio. No differences in IVS thickness, PW thickness, A-wave, E-wave, and E/A ratio were observed. Similarly, no significant differences in left ventricular mass were observed. Sac/val use was also associated with an average 5.1% increase in LVEF, from a mean baseline of 33.%1 + 2.1% to 38.2% + 3.0% (p < 0.0001) (**Table 4**). **Table 5** shows the differences occurring between the pharmacological treatments received at baseline and after 24 weeks of sac/val therapy. Notably, there was a significant reduction in diuretic drug use and a significant increase in lipid-lowering medication. Therapy with sac/val was well tolerated, and no serious adverse reactions occurred during the course of treatment. Regarding adverse events, there were only two episodes of hypotension, and only one required reduction of the dose of sac/val. Drug discontinuation has not been necessary for any patient. A transitory increase in the serum levels of potassium has been observed only in four patients. In addition, in two patients only, there was a worsening of HF that did not require hospitalization.

**Table 4:** Echocardiographic parameters at baseline and after 24 weeks of therapy with sac/val.

	Baseline	Follow-up	P Value
Variables	(n = 59)	(n = 59)	
**LAV**, mm	48.7 ± 5.4	46.1 ± 6.9	<0.05
**LVEDV**, ml	168 ± 24	158 ± 22	<0.05
**LVESV**, ml	111 ± 26	98 ± 23	<0.005
**LVEF**, %	33.1 ± 2.1	38.2 ± 3.0	<0.0001
**IVS thickness,** cm	1.1 ± 0.26	1.08 ± 0.25	0.6
**PW thickness,** cm	0.85 ± 0.10	0.83 ± 0.12	0.3
**E wave,** cm/s	0.69 ± 0.15	0.74 ± 0.27	0.2
**A wave**	0.78 ± 0.2	0.80 ± 0.4	0.7
**E/A ratio**	0.88 ± 0.2	0.92 ± 0.3	0.4
**E/**e′ **ratio**	9.7 ± 3.2	8.0 ± 2.7	<0.0002

Data are means ± SD.

LAV, left atrial volume; LVEDV, left ventricular end-diastolic volume; LVESV, left ventricular end-systolic volume; LVEF, left ventricular ejection fraction; IVS, interventricular septal; PW, posterior wall; sac/val, sacubitril/valsartan.

**Table 5:** Pharmacological treatments at baseline and after 24 weeks of therapy with sac/val.

	Baseline	Follow-up	P Value
Variables	(n = 59)	(n = 59)	
**ACE inhibitors**, n (%)	43 (73)	0 (0)0	<0.0001
**ARB**, n (%)	16 (27)	0 (0)	<0.0001
**MRA**, n (%)	27 (46)	25 (42)	0.7
**Loop diuretics**, n (%)	58 (98)	48 (81)	0.002
**Beta-blockers**, n (%)	57 (97)	57 (97)	0.99
**Antiplatelet agents**, n (%)	30 (51)	30 (51)	0.99
**Oral anticoagulants**, n (%)	19 (32)	19 (352)	0.99
**Statins**, n (%)	49 (83)	53 (90)	0.2
**Ezetimibe,** n (%)	0 (0)	9 (15)	<0.0001

ACE inhibitors, angiotensin-converting enzyme inhibitors; ARB, angiotensin receptor blockers; MRA, mineralocorticoid receptor antagonist; sac/val, sacubitril/valsartan.

Finally, 30 patients (51%) had an electronic device [implantable cardioverter defibrillator (ICD) or cardiac resynchronization therapy defibrillator (CRTd)]. These patients had been implanted at least 12 months prior to the start of sac/val treatment. In addition, patients who met the indication for CRTd or ICD implantation during the follow-up study would not have been considered for data analysis, by design. However, none of the enrolled patients met this indication.

## Discussion

In this study, we found that treatment with sac/val was able to improve both IR and metabolic profile in prediabetic non-obese patients with HFrEF. Until today, few data on metabolic changes in HFrEF patients without T2DM treated with sac/val were available.

As previously reported, in the *post-hoc* analysis of the PARADIGM-HF clinical study, in patients with T2DM, a greater reduction of HbA1c concentrations in the patients in treatment with sac/val than in those taking enalapril has been demonstrated. Furthermore, during the study, fewer participants in the sac/val group required initiation of insulin therapy or oral antihyperglycemic therapy for glycemic control as compared with the enalapril group. These data suggested that in addition to reducing the risk of cardiovascular death, hospital admission for heart failure, and all-cause mortality, sac/val treatment might have favorable metabolic effects in patients with HF and diabetes (9). However, IR is common also in non-diabetic patients with HF and is associated with adverse prognosis (8).

In our study, we found an improvement in HOMA-IR, fasting blood glucose, fasting insulin, and HbA1c in prediabetic patients. To evaluate insulin sensitivity, we used the HOMA model. Although the euglycemic–hyperinsulinemic clamp is considered the gold standard method to measure insulin sensitivity, it is time-consuming and expensive and is not feasible in large-scale studies. HOMA-IR is a robust and easy method for assessing β-cell function and insulin resistance (15).

There are several potential mechanisms by which the block of the RAAS and the inhibition of neprilysin might lead to improvement in metabolic parameters and insulin resistance. The benefits of RAAS inhibition may be due to its effects on both peripheral IR and insulin secretion. Angiotensin II and aldosterone are able to induce IR altering insulin signaling and leading to decreased glucose transport at the cellular level. Angiotensin II also contributes to inflammation, oxidative stress, and apoptosis in pancreatic β cells (16, 17). Several large clinical trials have demonstrated that the inhibition of RAAS reduced the incidence of diabetes in patients with HF and/or risk for coronary artery disease probably through different pathways including increased perfusion of insulin-sensitive muscles and tissues with improved cellular insulin sensitivity and increased insulin secretion (17–19).

Moreover, although the effects of RAAS inhibition on insulin secretion have not been studied extensively, several evidence suggests that RAAS affects insulin secretion in both animals and humans (20–22). Drugs that block the RAAS could reduce diabetes risk by preserving β-cell function. Renin inhibition increases glucose tolerance and insulin secretion in the genetically obese diabetic KK-A mice (23). Similarly, treatment with valsartan is able to improve IR and increase glucose-stimulated insulin secretion in patients with impaired fasting glucose or impaired glucose tolerance (24).

**Figure 1:**
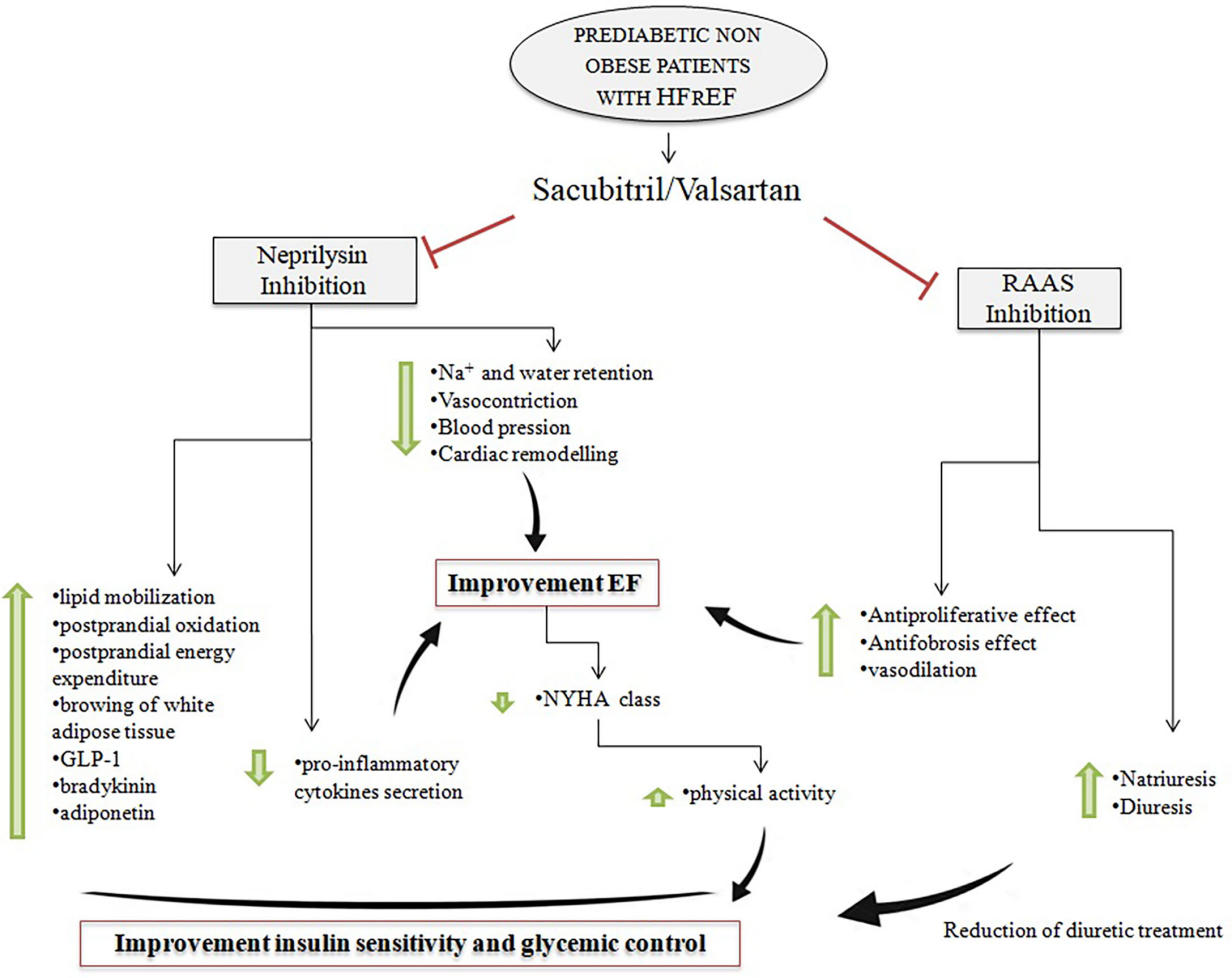
Effects of neprilysin inhibition and RAAS inhibition on insulin sensitivity and EF in prediabetic non-obese patients with HFrEF in treatment with sac/val. RAAS, renin–angiotensin–aldosterone system; EF, ejection fraction; HFrEF, heart failure with reduced ejection fraction; sac/val, sacubitril/valsartan.

Similarly, the inhibition of neprilysin might lead to improvement in glycemic control, IR, and metabolism through several potential mechanisms. Neprilysin is an enzyme that degrades several vasoactive peptides, including biologically active natriuretic peptides, angiotensin I and II, bradykinin, adrenomedullin, and glucagon-like peptide-1 (GLP-1) (25–27) and is expressed in many tissues [adipocytes, epithelial and smooth muscle cells, and endothelial and cardiac myocytes (28)]. Inhibition of neprilysin induces an increase of NPs that might have an important role in metabolism and insulin resistance. In particular, the increase of NP levels can favor lipid mobilization and postprandial oxidation with an increase in postprandial energy expenditure (2), increase adiponectin synthesis in the adipose tissue improving both IR and glucose metabolism (29), and promote white adipose tissue browning (30), all mechanisms that promote insulin sensitivity. In addition, the increase of NP levels can induce the inhibition of pro-inflammatory cytokines, such as interleukin-6 secretion (31). Therefore, by reducing interleukin-6 and increasing adiponectin levels from adipose tissue, NPs could enhance insulin sensitivity (32). Moreover, also the levels of other substances are modified by NEP inhibition; in particular, bradykinin and GLP-1 levels are increased, and endothelin-1 levels are decreased. Bradykinin may positively affect insulin signaling, glucose uptake, free fatty acid synthesis, and adiponectin expression in adipose tissue, improving insulin sensitivity and glycemic control (33, 34). Recently, it has been reported that the increase of NP levels is able to induce an augmentation of GLP-1 levels, a neuropeptide of the incretin family and potent antihyperglycemic hormone (35). GLP-1 may regulate insulin secretion and stimulate extrapancreatic glucoregulation; moreover, it may affect appetite and food intake. Finally, lower endothelin-1 levels are associated with reduced lipolysis and improved insulin sensitivity (36). In addition, the positive metabolic effects of sac/val treatment could be partly justified also by the improvement in quality of life and NYHA class, which could have favored an increase in physical activity and the reduction of diuretic drugs during the follow-up. We have no body composition measurements available, and we cannot know if ameliorating the NYHA class also improved body composition. However, it is plausible that the NYHA class improvement may increase the physical activity level of the patients, a well-known factor that affects insulin resistance. In addition, both the improvement of the NYHA class and the increase in physical activity level may modify body composition by increasing lean body mass, another factor that reduces insulin resistance. The effects of sac/val treatment in this group of patients are summarized in **Figure 1**.

According to previous studies (37–40), in our patients, we also observed that treatment with sac/val significantly improve left ventricular (LV) remodeling and function parameters. Sac/val treatment was associated with a reduction in LAV and end-systolic and end-diastolic LV volumes; together with this, also diastolic function parameters were improved, with an E/A ratio increase and E/e′ reduction, thus showing a lowering of intraventricular filling pressure. LV contractility was also ameliorated, as demonstrated by the significant change in LVEF.

Obviously, the reverse myocardial remodeling may justify the reduction in NT-proBNP levels that indicate a better hemodynamic condition and an improvement in both NYHA class and Minnesota Living with Heart Failure Questionnaire (MLHFQ) score observed in our patients.

Finally, the reduction in the use of loop diuretics may, at least in part, justify the important reduction in uric acid levels, a well-established cardiovascular risk factor, especially when associated with other metabolic abnormalities such as IR (41).

The present study has some limitations. First, this study has a small number of patients. Second, it is a pilot study from only two centers. Third, it is not a randomized clinical trial, and a matched control group is not available.

## Conclusions

In conclusion, our data suggest that in addition to the HF benefits previously demonstrated, sac/val might have favorable metabolic effects also in prediabetic patients with HF and without obesity. In our study, we demonstrated an improvement in metabolic profile and IR during treatment with sac/val. This evidence is important because hyperglycemia and insulin resistance facilitate atherosclerotic, inflammatory, and fibrosis processes and might have implications for the pathophysiology of HF disease progression. Thus, therapeutically targeting impaired insulin resistance and prediabetic condition could potentially be beneficial in patients with heart failure. However, studies with a higher number of participants are needed.

## Data availability statement

The raw data supporting the conclusions of this article will be made available by the authors, without undue reservation.

## Ethics statement

This study was reviewed and approved by Ethics Committee Regione Calabria (Area Centro)(protocol code code protocol number 2012.63). The patients/participants provided their written informed consent to participate in this study.

## Author contributions

Conceptualization: CoC, IZ, LS, FeA, CrC, SL, MCP, FG, GA, and VF. Methodology: AS and FrA. Validation: FMDR, AS, and FrA. Formal analysis: FrA. Investigation: CoC, IZ, LS, FeA, CrC, SL, MCP, FG, GA, and VF. Data curation: CoC, FMDR, AS, and FrA. Writing—original draft preparation: CoC, IZ, LS, FeA, CrC, SL, MCP, FG, GA, and VF: Writing—review and editing: FrA. Supervision: FMDR, AS, and FrA. Project administration: AS and FrA. All authors contributed to the article and approved the submitted version.

## Acknowledgments

We want to thank Prof. Hribal Marta Letizia for her valuable contribution to the revision of the English language.

## Conflict of interest

The authors declare that the research was conducted in the absence of any commercial or financial relationships that could be construed as a potential conflict of interest.

## Publisher’s note

All claims expressed in this article are solely those of the authors and do not necessarily represent those of their affiliated organizations, or those of the publisher, the editors and the reviewers. Any product that may be evaluated in this article, or claim that may be made by its manufacturer, is not guaranteed or endorsed by the publisher.

## Abbreviations

ARNI, angiotensin receptor-neprilysin inhibitor; BBs, beta-blockers; ECG, electrocardiogram; EF, ejection fraction; eGFR, estimated glomerular filtration rate; ESC, European Society of Cardiology; FPG, fasting plasma glucose; GLP-1, glucagon like peptide-1; RAAS, renin–angiotensin–aldosterone system; HbA1c, hemoglobin A1c; HF, heart failure; HDL-cholesterol, high-density lipoprotein cholesterol; HFpEF, heart failure with preserved ejection fraction; HFrEF, heart failure with reduced ejection fraction; HOMA, Homeostatic Model Assessment; IFG, impaired fasting glucose; IGT, impaired glucose tolerance; IR, insulin resistance; IVS, interventricular septum; LAV, left atrial volume; LDL-cholesterol, low-density lipoprotein cholesterol; LV, left ventricular; LVEDV, left ventricular end-diastolic volume; LVEF, left ventricular ejection fraction; LVESV, left ventricular end-systolic volume; LVID, left ventricular internal diameter; NGT, normal glucose tolerance; NPs, natriuretic peptides; NT-proBNP, N-terminal pro-brain natriuretic peptide; NYHA, New York Heart Association; OGTT, oral glucose tolerance test; PW, posterior wall; sac/val, sacubitril/valsartan; T2DM, type 2 diabetes mellitus.

## References

[1] Savarese G Lund LH. Global public health burden of heart failure. Card Fail Rev (2017) 3(1):7–11. doi: 10.15420/cfr.2016:25:2 28785469 PMC5494150

[2] Vardeny O Miller R. Combined neprilysin and renin-angiotensin system inhibition for the treatment of heart failure. JACC Heart Fail (2014) 2(6):663–70. doi: 10.1016/j.jchf.2014.09.001 25306450

[3] McMurray JJ Packer M. Angiotensin-neprilysin inhibition versus enalapril in heart failure. N Engl J Med (2014) 371(11):993–1004. doi: 10.1056/NEJMoa1409077 25176015

[4] Dunlay SM Givertz MM. Type 2 diabetes mellitus and heart failure: A scientific statement from the American heart association and the heart failure society of America: This statement does not represent an update of the 2017 ACC/AHA/HFSA heart failure guideline update [published correction appears in circulation. Circulation (20192019) 140(7):e294–324. doi: 10.1161/CIR.0000000000000691 31167558

[5] Pocock SJ Wang D. Predictors of mortality and morbidity in patients with chronic heart failure. Eur Heart J (2006) 27(1):65–75. doi: 10.1093/eurheartj/ehi555 16219658

[6] Paolillo S Rengo G. Insulin resistance is associated with impaired cardiac sympathetic innervation in patients with heart failure. Eur Heart J Cardiovasc Imag (2015) 16(10):1148–53. doi: 10.1093/ehjci/jev061 25845954

[7] Shimizu I Minamino T. Excessive cardiac insulin signaling exacerbates systolic dysfunction induced by pressure overload in rodents. J Clin Invest (2010) 120(5):1506–14. doi: 10.1172/JCI40096 PMC2860916 20407209

[8] Doehner W Rauchhaus M. Impaired insulin sensitivity as an independent risk factor for mortality in patients with stable chronic heart failure. J Am Coll Cardiol (2005) 46(6):1019–26. doi: 10.1016/j.jacc.2005.02.093 16168285

[9] Seferovic JP Claggett B. Effect of sacubitril/valsartan versus enalapril on glycaemic control in patients with heart failure and diabetes: a post-hoc analysis from the PARADIGM-HF trial. Lancet Diabetes Endocrinol (2017) 5(5):333–40. doi: 10.1016/S2213-8587(17)30087-6 PMC5534167 28330649

[10] American Diabetes Association. Introduction: Standards of medical care in diabetes–2022. Diabetes Care (2022) 45(Supplement_1):S1–2. doi: 10.2337/dc22-Sint 34964812

[11] Kularatna S Senanayake S Chen G Parsonage W. Mapping the Minnesota living with heart failure questionnaire (MLHFQ) to EQ-5D-5L in patients with heart failure. Health Qual Life Outcomes. (2020) 18(1):115. doi: 10.1186/s12955-020-01368-2 32349782 PMC7189529

[12] Ponikowski P Voors AA. 2016 ESC guidelines for the diagnosis and treatment of acute and chronic heart failure: The task force for the diagnosis and treatment of acute and chronic heart failure of the European society of cardiology (ESC). developed with the special contribution of the heart failure association (HFA) of the ESC. Eur J Heart Fail (2016) 18(8):891–975. doi: 10.1002/ejhf.592 27207191

[13] Lang RM Badano LP. Recommendations for cardiac chamber quantification by echocardiography in adults: an update from the American society of echocardiography and the European association of cardiovascular imaging. Eur Heart J Cardiovasc Imaging (2015) 16(3):233–70. doi: 10.1093/ehjci/jev014 25712077

[14] Nagueh SF Smiseth OA. Recommendations for the evaluation of left ventricular diastolic function by echocardiography: An update from the American society of echocardiography and the European association of cardiovascular imaging. Eur Heart J Cardiovasc Imag (2016) 17(12):1321–60. doi: 10.1093/ehjci/jew082 27422899

[15] Matthews DR Hosker JP. Homeostasis model assessment: insulin resistance and beta-cell function from fasting plasma glucose and insulin concentrations in man. Diabetologia (1985) 28(7):412–9. doi: 10.1007/BF00280883 3899825

[16] Underwood PC Adler GK. The renin angiotensin aldosterone system and insulin resistance in humans. CurrHyper Rep (2013) 15(1):59–70. doi: 10.1007/s11906-012-0323-2 PMC3551270 23242734

[17] Luther JM Brown NJ. The renin-angiotensin-aldosterone system and glucose homeostasis. Trends Pharmacol Sci (2011) 32(12):734–9. doi: 10.1016/j.tips.2011.07.006 PMC3223326 21880378

[18] Scheen AJ. Prevention of type 2 diabetes mellitus through inhibition of the renin-angiotensin system. Drugs (2004) 64(22):2537–65. doi: 10.2165/00003495-200464220-00004 15516153

[19] NAVIGATOR Study Group McMurray JJ Holman RR. Effect of valsartan on the incidence of diabetes and cardiovascular events. N Engl J Med (2010) 362(16):1477–90. doi: 10.1056/NEJMoa1001121 20228403

[20] Yuan L Li X. Effects of renin-angiotensin system blockade on islet function in diabetic rats. J Endocrinol Invest (2010) 33(1):13–9. doi: 10.1007/BF03346544 20203537

[21] Luther JM Luo P. Aldosterone decreases glucose-stimulated insulin secretion *in vivo* in mice and in murine islets. Diabetologia (2011) 54(8):2152–63. doi: 10.1007/s00125-011-2158-9 PMC3216479 21519965

[22] Fliser D Schaefer F. Angiotensin II affects basal, pulsatile, and glucose-stimulated insulin secretion in humans. Hypertension (1997) 30(5):1156–61. doi: 10.1161/01.HYP.30.5.1156 9369270

[23] Iwai M Kanno H. Direct renin inhibition improved insulin resistance and adipose tissue dysfunction in type 2 diabetic KK-a(y) mice. J Hypertens (2010) 28(7):1471–81. doi: 10.1097/HJH.0b013e32833bc420 20543712

[24] van der Zijl NJ Moors CC. Valsartan improves {beta}-cell function and insulin sensitivity in subjects with impaired glucose metabolism: a randomized controlled trial. Diabetes Care (2011) 34(4):845–51. doi: 10.2337/dc10-2224 PMC3064038 21330640

[25] Kobalava Z Kotovskaya Y Averkov O Pavlikova E Moiseev V Albrecht D. Pharmacodynamic and pharmacokinetic profiles of sacubitril/valsartan (LCZ696) in patients with heart failure and reduced ejection fraction. Cardiovasc Ther (2016) 34(4):191–8. doi: 10.1111/1755-5922.12183 PMC5108443 26990595

[26] Cruden NL Fox KA. Neutral endopeptidase inhibition augments vascular actions of bradykinin in patients treated with an-giotensin-converting enzyme inhibition. Hypertension (2004) 44(6):913–8. doi: 10.1161/01.HYP.0000146483.78994.56 15492133

[27] Wilkinson IB McEniery CM. Adrenomedullin (ADM) in the human forearm vascular bed: effect of neutral endopeptidase inhibition and comparison with proadrenomedullin NH2-terminal 20 peptide (PAMP). Br J Clin Pharmacol (2001) 52(2):159–64. doi: 10.1046/j.0306-5251.2001.1420.x PMC2014526 11488772

[28] Turner AJ Isaac RE Coates D. The neprilysin (NEP) family of zinc metalloendopeptidases: genomics and function. Bioessays (2001) 23(3):261–9. doi: 10.1002/1521-1878(200103)23:3<261::AID-BIES1036>3.0.CO;2-K 11223883

[29] Tsukamoto O Fujita M. Natriuretic peptides enhance the production of adiponectin in human adipocytes and in patients with chronic heart failure. J Am Coll Cardiol (2009) 53(22):2070–7. doi: 10.1016/j.jacc.2009.02.038 19477358

[30] Bordicchia M Liu D. Cardiac natriuretic peptides act *via* p38 MAPK to induce the brown fat thermogenic program in mouse and human adipocytes. J Clin Invest (2012) 122(3):1022–36. doi: 10.1172/JCI59701 PMC3287224 22307324

[31] Moro C Klimcakova E. Atrial natriuretic peptide inhibits the production of adipokines and cytokines linked to inflammation and insulin resistance in human subcutaneous adipose tissue. Diabetologia (2007) 50(5):1038–47. doi: 10.1007/s00125-007-0614-3 17318625

[32] Seferovic JP Solomon SD. Potential mechanisms of beneficial effect of sacubitril/valsartan on glycemic control. Ther Adv Endocrinol Metab (2020) 11:2042018820970444. doi: 10.1177/2042018820970444 33489085 PMC7768573

[33] Beard KM Lu H. Bradykinin augments insulin-stimulated glucose transport in rat adipocytes *via* endothelial nitric oxide synthase-mediated inhibition of jun NH2-terminal kinase. Diabetes (2006) 55(10):2678–87. doi: 10.2337/db05-1538 17003331

[34] Shiuchi T Cui TX. ACE inhibitor improves insulin resistance in diabetic mouse *via* bradykinin and NO. Hypertension (2002) 40(3):329–34. doi: 10.1161/01.HYP.0000028979.98877.0C 12215475

[35] Vodovar N Nougué H. Sacubitril/valsartan in PARADIGM-HF. Lancet Diabetes Endocrinol (2017) 5(7):495–6. doi: 10.1016/S2213-8587(17)30177-8 28645438

[36] Eriksson AK van Harmelen V. Endothelin-1 stimulates human adipocyte lipolysis through the ET a receptor. Int J Obes (Lond). (2009) 33(1):67–74. doi: 10.1038/ijo.2008.212 18982011

[37] Almufleh A Marbach J. Ejection fraction improvement and reverse remodeling achieved with Sacubitril/Valsartan in heart failure with reduced ejection fraction patients. Am J Cardiovasc Dis (2017) 7(6):108–13. PMC5768868 29348971

[38] Bayard G Da Costa A. Impact of sacubitril/valsartan on echo parameters in heart failure patients with reduced ejection fraction a prospective evaluation [published correction appears in int J cardiol heart vasc. 2020 Dec 19;32:100698]. Int J Cardiol Heart Vasc (2019) 25:100418. doi: 10.1016/j.ijcha.2019.100418 PMC6728827 31517034

[39] Carluccio E Dini FL. Benefit from sacubitril/valsartan is associated with hemodynamic improvement in heart failure with reduced ejection fraction: An echocardiographic study. Int J Cardiol (2022) 350:62–8. doi: 10.1016/j.ijcard.2022.01.004 34998946

[40] Armentaro G D’Arrigo G. Impact of Sacubitril/Valsartan on clinical and echocardiographic parameters in heart failure patients with reduced ejection fraction: Data from a real life 2-year follow-up study. Front Pharmacol (2021) 12:733475. doi: 10.3389/fphar.2021.733475 34483943 PMC8415264

[41] Cassano V Crescibene D. Uric acid and vascular damage in essential hypertension: Role of insulin resistance. Nutrients (2020) 12(9):2509. doi: 10.3390/nu12092509 PMC7551393 32825165

